# The Triglyceride–Glucose Index and Colorectal Adenoma: A CHungcheong Association for the Study of Intestinal Disease (CHASID) Multi-Center Cross-Sectional Study

**DOI:** 10.3390/jcm15135147

**Published:** 2026-07-02

**Authors:** Dae Sung Kim, Hoon Sup Koo, Sanghyuk Lee, Jeong Eun Shin, Yunho Jung, Sang-Bum Kang, Hee Seok Moon, Won Kang Jeong, Sung Bin Park, Kyu Chan Huh

**Affiliations:** 1 Division of Gastroenterology, Department of Internal Medicine, Konyang University College of Medicine, Daejeon 35365, Republic of Korea; hornkim@kyuh.ac.kr (D.S.K.); koo7574@kyuh.ac.kr (H.S.K.); jeschi@kyuh.ac.kr (S.L.); 200823@kyuh.ac.kr (W.K.J.); 2 Myunggok Medical Research Center, Konyang University College of Medicine, Daejeon 35365, Republic of Korea; 3 Division of Gastroenterology, Department of Internal Medicine, Dankook University College of Medicine, Cheonan 31116, Republic of Korea; dreun@dankook.ac.kr; 4 Division of Gastroenterology, Department of Internal Medicine, Soonchunhyang University College of Medicine, Cheonan 31151, Republic of Korea; c73138@schmc.ac.kr; 5 Division of Gastroenterology, Department of Internal Medicine, Daejeon St. Mary’s Hospital, College of Medicine, The Catholic University of Korea, Daejeon 34943, Republic of Korea; sangucsd@gmail.com; 6 Division of Gastroenterology, Department of Internal Medicine, Chungnam National University School of Medicine, Daejeon 35015, Republic of Korea; mhs1357@cnuh.co.kr; 7 Department of Medicine, Samsung Medical Center, Sungkyunkwan University School of Medicine, Seoul 06351, Republic of Korea

**Keywords:** triglyceride–glucose index, colorectal adenoma, insulin resistance, colonoscopy, screening, metabolic syndrome, risk stratification

## Abstract

**Background/Objectives**: Insulin resistance is increasingly recognized as a cause of colorectal neoplasms, but its measurement requires fasting insulin, which is not routinely available in clinical settings. The triglyceride–glucose (TyG) index, derived from fasting triglyceride and glucose, has emerged as a simple surrogate of insulin resistance. We aimed to evaluate the association of the TyG index with colorectal adenoma, identify a clinically usable cut-off, and examine whether the association is preserved across major subgroups. **Methods:** We conducted a cross-sectional analysis of 7251 asymptomatic adults who underwent screening colonoscopy and same-day biochemistry at university hospital health care centers in Daejeon and Chungcheong province of South Korea between November 2019 and June 2022. The TyG index was calculated as ln[fasting triglycerides (mg/dL) × fasting glucose (mg/dL)/2]. Multivariable logistic regression was used to estimate odds ratios (ORs) for adenoma; discrimination was evaluated by area under the receiver-operating-characteristic curve (AUC), and the optimal cut-off was identified by Youden’s J. Large adenoma (≥10 mm) was analyzed as a secondary outcome. **Results:** Among 7251 participants (mean age 54.1 ± 11.2 years; 59.7% male; mean BMI 24.7 ± 3.4 kg/m^2^), 2402 (33.1%) had at least one colorectal adenoma. Adenoma prevalence rose monotonically across TyG quartiles (Q) (Q1, 26.3%; Q2, 32.0%; Q3, 35.5%; Q4, 38.7%; *p* for trend <0.001). A 1-standard deviation (SD) increase in TyG index was associated with adenoma prevalence in the fully adjusted model (OR 1.13, 95% confidence interval (CI) 1.06–1.20), and the Q4-versus-Q1 OR was 1.29 (1.09–1.53). The optimal cut-off for adenoma was TyG index = 8.55 (AUC 0.564, sensitivity 59.1%, specificity 50.8%); the association was modestly stronger for large adenoma (AUC 0.585; adjusted OR per 1-SD 1.25, 1.09–1.43). Subgroup analyses showed consistent effects across sex, age, body mass index, hypertension, diabetes, and metabolic-syndrome strata (all *p* for interaction >0.17). **Conclusions:** In a large screening cohort, an elevated TyG index was associated with the presence of colorectal adenoma, with a graded dose–response relationship and a modestly more pronounced association for large adenoma. Although discrimination by TyG index alone is too modest to support its use as a stand-alone screening tool, the index may serve as a low-cost adjunct within a multifactorial risk-stratification framework.

## 1. Introduction

Colorectal cancer (CRC) remains a leading cause of cancer-related morbidity and mortality worldwide, with an estimated 1.93 million new cases and 0.94 million deaths in 2020, and a projected continued rise through 2040 [1]. Most CRCs arise from precursor adenomatous polyps through the well-established adenoma–carcinoma sequence [2,3], and the detection and removal of adenomas during colonoscopy reduces incident CRC and CRC-related mortality [4,5]. Adenoma detection rate is consequently a validated quality indicator that is inversely associated with interval CRC and CRC death [6,7].

Despite the well-defined morphologic pathway, the systemic metabolic background that promotes adenoma formation is incompletely characterized. Accumulating evidence implicates insulin resistance and the broader metabolic syndrome in colorectal carcinogenesis [8,9,10,11]. Hyperinsulinemia drives mitogenic signaling through the insulin/insulin-like growth factor-1 axis and downstream phosphoinositide 3-kinase/protein kinase B (PI3K/Akt) and mitogen-activated protein kinase pathways, modulating epithelial proliferation, apoptosis, and adenoma progression [12,13]. Case–control and cohort studies have consistently linked components of the metabolic syndrome, hyperglycemia, dyslipidemia, and hypertension to increased adenoma and CRC risk [14,15,16], and a meta-analysis of metabolic-syndrome studies estimated a 25% higher CRC risk in women and a 33% higher risk in men among affected individuals [10].

Established surrogates of insulin resistance, such as the homeostatic model assessment of insulin resistance (HOMA-IR), require a fasting insulin measurement that is not part of routine health-screening panels. The triglyceride–glucose (TyG) index, calculated as ln[fasting triglycerides (mg/dL) × fasting glucose (mg/dL)/2], has been proposed as a simple, low-cost surrogate that is computable from standard biochemistry [17,18]. The TyG index correlates well with HOMA-IR and has shown utility as a predictor of incident hypertension [19], cardiovascular events [20], and coronary artery calcification [21].

Recent epidemiologic studies have begun to extend the TyG index into an oncologic risk prediction tool. Liu et al., using prospective cohort data, reported a positive association of the TyG index with incident CRC risk [22], and Okamura et al. found that a higher TyG index predicted incident CRC during a median 4.4-year follow-up in a Japanese cohort [23]. More recently, Zhu et al. observed a graded association between the TyG index and the presence of colorectal adenoma in 1538 asymptomatic Asian adults [24]. However, data linking the TyG index to adenoma prevalence in large screening populations remain limited, the discriminative ability of the TyG index for adenoma has not been quantified in detail, and an actionable cut-off for clinical application is not established.

Established risk factors for colorectal adenoma include older age, male sex, family history of colorectal neoplasia, obesity, smoking, alcohol intake, diabetes, dyslipidemia, and physical inactivity [3]; insulin resistance is increasingly recognized as a potentially modifiable metabolic contributor to this risk pattern. To address these gaps, we conducted a cross-sectional study of 7251 asymptomatic adults who underwent screening colonoscopy and same-day biochemistry at university hospital health-screening centers. We aimed to (1) characterize the independent association between the TyG index and colorectal adenoma after adjustment for established risk factors; (2) determine the shape of the dose–response relationship; (3) identify and evaluate an optimal TyG cut-off for adenoma prediction; and (4) examine whether the association is preserved across pre-specified clinical subgroups.

## 2. Materials and Methods

### 2.1. Study Design and Participants

We performed a multi-center, cross-sectional study of consecutive adults who underwent a comprehensive health-screening examination, including colonoscopy and same-day fasting biochemistry, at university hospital health screening centers in Daejeon and Chungcheong province of South Korea between November 2019 and June 2022. Participants with findings suggestive of or a confirmed diagnosis of inflammatory bowel disease on endoscopy and those with suspected or confirmed colorectal cancer were excluded from the study in advance. After application of these criteria, 7251 participants with complete TyG index, laboratory tests, colonoscopic information formed the cohort for analysis.

The study was conducted in accordance with the Declaration of Helsinki and approved by the Institutional Review Board of Konyang university hospital (protocol code KYUH 2022-12-027, date of approval 4 January 2023). Because of the retrospective design and the de-identification of all records, the requirement for individual informed consent was waived by the institutional review board.

### 2.2. Colonoscopy and Histologic Assessment

All colonoscopies were performed by board-certified gastroenterologists using standard high-definition white-light endoscopes. Most polyps requiring resection were removed using biopsy forceps, a cold snare, endoscopic mucosal resection, or endoscopic submucosal dissection, depending on the situation, and the specimens were submitted for histopathological examination. For polyps that could not be removed during a health screening due to issues such as size or shape, a biopsy was performed to confirm the histological findings, or the patient was referred to the gastroenterology department at the same institution to undergo a colonoscopic procedure. The primary outcome was the presence of any colorectal adenoma, defined histologically as tubular adenoma, tubulovillous adenoma, villous adenoma, traditional serrated adenoma, or sessile serrated adenoma. We acknowledge that “sessile serrated adenoma” represents historic terminology that substantially overlaps with the current World Health Organization “sessile serrated lesion” nomenclature; we therefore consider this category at risk of misclassification within the serrated pathway. Hyperplastic polyps and lesions classified as sessile serrated lesion under current World Health Organization terminology were not included as adenomas. The size of the largest adenoma per participant was recorded; large adenoma (≥10 mm) was analyzed as a secondary outcome as a clinically meaningful surrogate for advanced adenoma, given that histologic descriptors of villous component and high-grade dysplasia were not consistently available in the dataset.

### 2.3. Biochemistry and Anthropometric Measurements

After an overnight fast of at least 8 h, venous blood was drawn on the day of colonoscopy. Fasting plasma glucose, total cholesterol, triglyceride (TG), high-density lipoprotein cholesterol (HDL-C), low-density lipoprotein cholesterol (LDL-C), glycated hemoglobin (HbA1c), aspartate and alanine aminotransferase, blood urea nitrogen, creatinine, and complete blood counts were measured. The TyG index was calculated as ln[fasting TG (mg/dL) × fasting glucose (mg/dL)/2] [16]. Body mass index (BMI) was calculated as weight in kilograms divided by squared height in meters. Hypertension was defined as systolic blood pressure ≥ 130 mmHg or diastolic blood pressure ≥ 85 mmHg, in accordance with the modified national cholesterol education program (NCEP) criteria for the metabolic syndrome. Diabetes mellitus was defined as HbA1c ≥ 6.5%, and metabolic syndrome was assigned according to the harmonized national cholesterol education program adult treatment panel III (NCEP-ATP III) criteria with Asian-specific cut-offs.

### 2.4. Statistical Analysis

Continuous variables are presented as mean ± standard deviation (SD) for approximately normally distributed variables or as median (interquartile range, IQR) for skewed variables; categorical variables are presented as counts and percentages. Between-group comparisons of baseline characteristics used independent *t*-test for normally distributed continuous variables, the Mann–Whitney U test for skewed continuous variables, and Pearson’s chi-square test for categorical variables.

Adenoma prevalence was organized by TyG index quartiles, with the Cochran–Armitage trend test used to evaluate monotonic increase. Logistic regression models estimated the OR of adenoma per 1-standard deviation (SD) increase in TyG and across TyG quartiles (with Q1 as the reference), under four pre-specified models with sequential confounder adjustment: Model 1 (crude); Model 2 (Model 1 + age and sex); Model 3 (Model 2 + BMI and hypertension); and Model 4 (Model 3 + HDL-C, LDL-C, and diabetes). Identical models were fit for the secondary outcome of large adenoma (≥10 mm).

Discrimination of the TyG index for adenoma was quantified by the area under the receiver-operating-characteristic curve (AUC). Confidence intervals (CIs) for the AUC were calculated by the DeLong method with a logit-transform to constrain the bounds. The optimal cut-off was defined by maximization of Youden’s J index.

Subgroup analyses pre-specified by sex, age (<50 vs. ≥50 years), BMI (<25 vs. ≥25 kg/m^2^), hypertension status, diabetes status, and metabolic-syndrome status were performed using fully adjusted logistic regression within each stratum, with *p* for interaction estimated from the Wald test of the TyG × stratum cross-product term in the full-sample model. All tests were two-sided with α = 0.05.

The authors declare that generative AI technology was used to assist with data analysis and graphic generation. However, all study data were collected by the authors, who also conducted the literature review and wrote the manuscript. The authors remain fully responsible for the content and integrity of the final work.

## 3. Results

### 3.1. Baseline Characteristics

Among 7251 participants, 2402 (33.1%) had at least one colorectal adenoma. Baseline characteristics by adenoma status are summarized in Table 1. Compared with the no-adenoma group, participants with adenoma were older (mean age 57.3 ± 10.0 vs. 52.5 ± 11.5 years), more often male (67.7% vs. 55.7%), and had higher BMI, fasting plasma glucose, HbA1c, and triglyceride levels (all *p* < 0.001). The prevalence of hypertension (63.0% vs. 54.6%), prediabetes (38.4% vs. 34.4%), diabetes (16.7% vs. 10.2%), low HDL-C (16.8% vs. 13.5%), hypertriglyceridemia (30.0% vs. 24.5%), and metabolic syndrome (36.9% vs. 27.1%) was higher in the adenoma group (all *p* < 0.001). The mean TyG index was 8.72 ± 0.61 in the adenoma group versus 8.59 ± 0.60 in controls (*p* < 0.001).

### 3.2. TyG Quartile and Adenoma Prevalence

Across ascending TyG quartiles (Q1 < 8.19, Q2 8.19–8.59, Q3 8.59–9.01, Q4 ≥ 9.01), adenoma prevalence rose monotonically (Q1, 26.3%; Q2, 32.0%; Q3, 35.5%; Q4, 38.7%; *p* for trend < 0.001) (Table 2). The prevalence of large adenoma (≥10 mm) followed the same gradient (2.3%, 4.1%, 3.9%, and 5.4% across quartiles; *p* for trend <0.001). Higher TyG quartiles were paralleled by progressively higher mean BMI and by higher prevalence of hypertension, diabetes, and metabolic syndrome; the prevalence of metabolic syndrome reached 73.1% in Q4 versus 4.9% in Q1.

**Figure 1:**
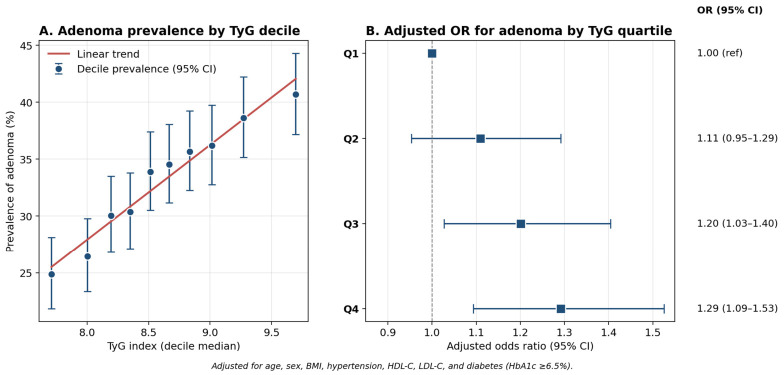
Dose–response relationship between the TyG index and the presence of colorectal adenoma. (**A**) Adenoma prevalence across TyG deciles (95% confidence intervals shown by error bars; red line shows the linear trend). (**B**) Adjusted odds ratios for adenoma by TyG quartile (Q1 reference), with 95% confidence intervals from the fully adjusted model.

Decile-level analysis demonstrated a clear graded dose–response relationship: adenoma prevalence increased step-wise from 24.9% in the lowest decile to 40.7% in the highest decile (Figure 1A), with a near-linear trend across deciles.

### 3.3. Univariable and Multivariable Logistic Regression

In univariable analysis (Table 3), the TyG index was significantly associated with adenoma both as a continuous variable (per 1-SD increase OR 1.25, 95% CI 1.19–1.31; *p* < 0.001) and as a quartile variable (Q4 vs. Q1 OR 1.78, 95% CI 1.54–2.05; *p* < 0.001). Age, male sex, BMI, hypertension, fasting plasma glucose, HbA1c, diabetes, triglyceride, low HDL-C, and metabolic syndrome were all univariable predictors of adenoma; LDL-C showed modest inverse association.

Multivariable logistic regression with stepwise confounder adjustment is shown in Table 4. After adjustment for age and sex (Model 2), the OR for TyG per 1-SD was 1.20 (1.14–1.27). After further adjustment for BMI and hypertension (Model 3) and for HDL-C, LDL-C, and diabetes (Model 4, the fully adjusted model), the OR remained significant at 1.13 (1.06–1.20; *p* < 0.001). The quartile-level adjusted ORs were 1.11 (0.95–1.29) for Q2, 1.20 (1.03–1.40) for Q3, and 1.29 (1.09–1.53) for Q4 versus Q1 (Figure 1B), confirming a graded independent association.

For the secondary outcome of large adenoma (≥10 mm), the fully adjusted OR per 1-SD increase in TyG was 1.25 (1.09–1.43; *p* = 0.001) (Table 5), indicating a more pronounced effect than for adenoma overall.

### 3.4. Discrimination and Optimal Cut-Off

**Figure 2:**
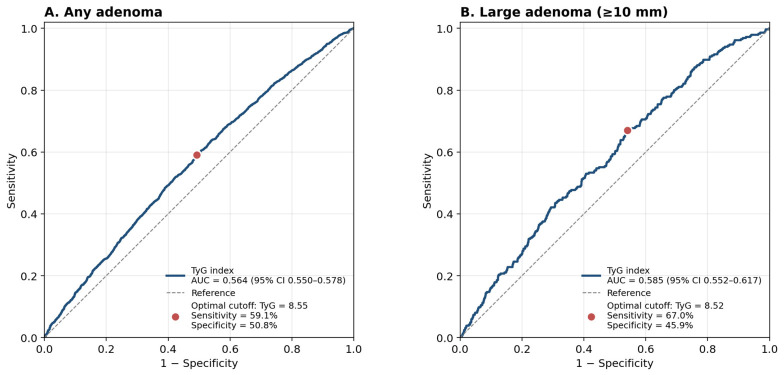
Receiver operating characteristic (ROC) curves for the TyG index in predicting colorectal adenoma. (**A**) Any adenoma; (**B**) large adenoma (≥10 mm). The red dot marks the Youden-optimal cut-off (J = sensitivity + specificity − 1). AUC, area under the curve; CI, confidence interval.

The AUC of the TyG index for any adenoma was 0.564 (95% CI 0.550–0.578) (Figure 2A). The Youden-optimal cut-off was TyG = 8.55, yielding sensitivity of 59.1% and specificity of 50.8% (Youden’s J = 0.099). For large adenoma, the AUC was 0.585 (95% CI 0.552–0.617), with an optimal cut-off of TyG = 8.52 (sensitivity 67.0%, specificity 45.9%; J = 0.129) (Figure 2B). At the optimal cut-off (TyG = 8.55), the positive and negative predictive values for any adenoma were 37.3% and 71.5%, respectively, reflecting the moderate prevalence of adenoma in this screening cohort.

### 3.5. Subgroup Analyses

**Figure 3:**
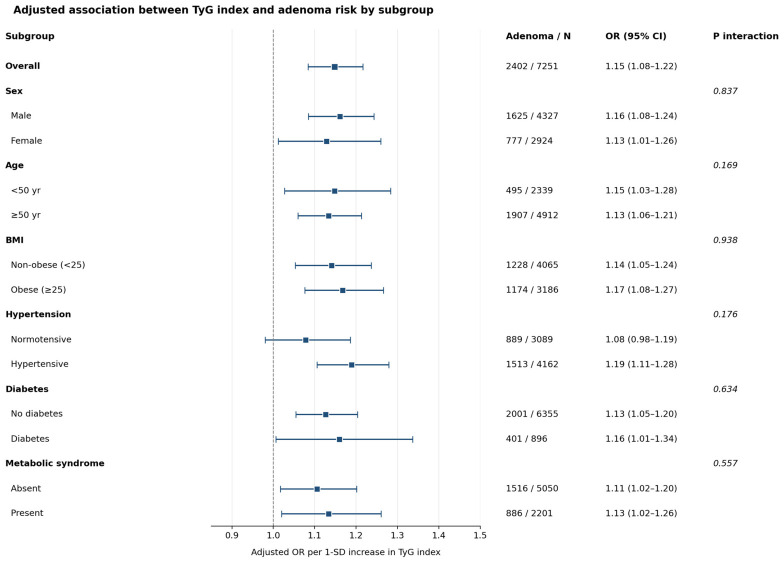
Adjusted association between the TyG index and the presence of colorectal adenoma within pre-specified subgroups. Each adjusted OR is per 1-SD increase in TyG, from logistic regression adjusted for age, sex, BMI, hypertension, diabetes and metabolic syndrome. *p* for interaction is from the Wald test of the TyG × stratum cross-product.

The fully adjusted association between TyG and adenoma was consistent across all pre-specified subgroups (Figure 3, Table 6). The adjusted OR per 1-SD increase ranged from 1.08 to 1.19 across strata, and none of the interaction tests reached statistical significance (*p* for interaction: sex 0.84, age 0.17, BMI 0.94, hypertension 0.18, diabetes 0.63, metabolic syndrome 0.56). Notably, the association persisted within both diabetic (OR 1.16, 1.01–1.34) and non-diabetic (OR 1.13, 1.05–1.20) strata, as well as within metabolic-syndrome-positive and -negative strata (OR 1.13 vs. 1.11). This consistency indicates that the TyG–adenoma association is not confined to subjects with diagnosed diabetes or metabolic syndrome; however, it does not by itself establish that the TyG index provides independent predictive information beyond these established conditions in a clinically meaningful way.

## 4. Discussion

In this cross-sectional analysis of 7251 asymptomatic adults undergoing screening colonoscopy, an elevated TyG index was independently associated with the presence of colorectal adenoma. The association persisted after adjustment for age, sex, BMI, hypertension, HDL-C, LDL-C, and diabetes, exhibited a graded dose–response relationship across the distribution of TyG, was more pronounced for large adenoma (≥10 mm), and was consistent across major demographic and metabolic subgroups. The Youden-optimal cut-off for adenoma was TyG = 8.55, although discrimination by TyG alone was modest (AUC 0.564 for any adenoma, 0.585 for large adenoma).

Our findings contribute to a growing body of evidence linking the TyG index with colorectal neoplasia. In a longitudinal Japanese cohort of 27,944 adults, Okamura et al. observed that higher TyG independently predicted incident CRC over a median of 4.4 years [23]. Liu et al. reported a positive association between TyG and prevalent CRC [22]. Most directly comparable, Zhu et al. recently described a graded TyG–adenoma relationship in 1538 asymptomatic Asians, with a particularly strong association for multiple adenomas (adjusted OR 1.74, 95% CI 1.17–2.57) [24]; adenoma multiplicity was not available as a secondary outcome in our dataset. Our results extend these observations by providing the largest cohort estimate of the TyG–adenoma association to date (n = 7251), by demonstrating that the association is robust to multivariable adjustment and homogeneous across clinically relevant subgroups, and by quantifying discriminative performance and a candidate cut-off. A recent systematic review and meta-analysis likewise reported a consistent positive association between elevated TyG index and incident CRC across observational studies, although the pooled discrimination remained modest [25].

Insulin resistance and compensatory hyperinsulinemia activate the insulin/insulin-like growth factor-1 receptor axis, stimulating colonic epithelial proliferation, impairing apoptosis, and promoting angiogenesis and chronic low-grade inflammation [12,26,27]. Insulin and insulin-like growth factor-1 have been associated with both presence and progression of adenomatous polyps in prospective data [26]. The TyG index integrates fasting hyperglycemia and atherogenic hypertriglyceridemia, which represent key downstream manifestations of hepatic and adipose tissue insulin resistance, thereby encapsulating a distinct metabolic phenotype mechanistically linked to colorectal carcinogenesis [17,18,28]. Consistent with this, individual components of the metabolic syndrome are known adenoma risk factors [14,29,30], yet our subgroup analyses indicate that the effect of TyG is not restricted to metabolic syndrome or diabetic individuals, suggesting that the index may captures a continuum of insulin resistance with adenoma relevance below clinical diagnostic thresholds.

The clinical utility of the TyG index for adenoma risk stratification deserves cautious interpretation. The discriminative performance for adenoma overall (AUC 0.564) is too modest to support the TyG index as a stand-alone screening tool; discrimination for large adenoma was only slightly better (AUC 0.585). These values are comparable to those reported for other single insulin-resistance surrogates (HOMA-IR, TG/HDL-C ratio) in adenoma and CRC prediction, and indicate that a single biomarker is unlikely to provide clinically actionable individual-level prediction. Rather than as a stand-alone diagnostic tool, the TyG index may be more appropriately considered as one of several inputs to multifactorial risk stratification, where its zero incremental cost and computability from routine fasting biochemistry are practical advantages compared with HOMA-IR. Subjects in the highest TyG quartile (≥9.01) had approximately 29% higher adjusted odds of adenoma and roughly 1.5-fold higher prevalence (38.7% vs. 26.3%) compared with the lowest quartile, which is biologically informative but should not be over-interpreted as a clinically decisive gradient. Beyond colorectal adenoma, a large pooled European analysis (Fritz et al., n = 510,471) reported that the TyG index was associated with several obesity-related cancers and substantially mediated the BMI–digestive-tract-cancer relationship [31], supporting the broader concept that insulin resistance, as captured by the TyG index, may partly underlie obesity-related digestive carcinogenesis. Although our cross-sectional analysis did not include formal mediation testing, the persistence of the TyG–adenoma association after adjustment for BMI in our fully adjusted model is directionally consistent with this mediation framework.

This study has several limitations. First, our adenoma case definition partially overlaps with the serrated-pathway terminology in evolution: lesions coded under the historic “sessile serrated adenoma” nomenclature were retained as adenomas, whereas lesions coded under the current “sessile serrated lesion” (SSL) nomenclature were excluded. The relative proportion of cases coded under each nomenclature could not be retrospectively retrieved from our dataset. However, recent published data indicate that the overall detection rate of SSL during average-risk screening colonoscopy is modest: a meta-analysis of 280,370 procedures reported a pooled SSL detection rate of approximately 2.5% [32], and a comprehensive review cited SSL prevalence estimates of 2–8% across centers, with SSL with cytological dysplasia accounting for only 0.4–0.6% of screening colonoscopies [33]. Given this low base rate of SSL relative to the high prevalence of conventional adenomas, the magnitude of any misclassification effect on our quartile-level estimates is expected to be small, although it cannot be entirely excluded. Second, the histologic descriptors of villous component and high-grade dysplasia were not consistently available, precluding application of the formal advanced-adenoma definition; we therefore used ≥10 mm size as a clinically meaningful surrogate. Third, the cross-sectional design precludes causal inference and risks reverse causation. Fourth, several major adenoma-related variables, including smoking, alcohol consumption, dietary patterns, physical activity, family history of colorectal neoplasia, and the use of aspirin, statins, or metformin, were not consistently recorded across the participating centers. We regard the absence of these variables as a major source of residual confounding rather than a minor caveat, and the multivariable estimates should be interpreted as adjusted for the measured covariates only. Fifth, all participating centers were located in the Daejeon and Chungcheong region of South Korea, and participants were individuals self-selecting for comprehensive health screening. The cohort therefore represents a relatively health-conscious, urban, ethnically homogeneous Korean population that may differ from average-risk screening populations elsewhere with respect to health behavior, socioeconomic profile, and referral patterns. The findings should be replicated in independent and ethnically diverse cohorts before generalization to broader screening settings.

Our study also has notable strengths. The sample size (n = 7251) is substantially larger than that of comparable studies, providing precise estimates and adequate power for subgroup analyses. All adenomas were histologically confirmed, eliminating recall or imaging bias. The use of stepwise multivariable adjustment, a pre-specified subgroup plan with formal interaction testing, and analysis of large adenoma as a clinically meaningful secondary outcome together yield results that are internally consistent and clinically interpretable.

## 5. Conclusions

In a large screening cohort of asymptomatic adults, the TyG index was associated with the presence of colorectal adenoma in a graded, dose-dependent manner. The association was preserved after multivariable adjustment, consistent across major subgroups, and modestly more pronounced for large adenoma. Although discrimination by the TyG index alone is insufficient to support its use as a stand-alone screening test, the index—computable from routine fasting biochemistry at no incremental cost—may be a useful adjunct within multifactorial adenoma risk-stratification frameworks. Prospective longitudinal studies are required to determine whether incorporating the TyG index into composite risk scores improves colonoscopy targeting and clinical outcomes.

## Figures and Tables

**Table 1:** Baseline characteristics by colorectal adenoma status.

Variable	No Adenoma (n = 4849)	Adenoma (n = 2402)	*p*-Value
Age, years	52.48 ± 11.53	57.28 ± 9.96	<0.001
Sex, n (%)			<0.001
Male	2702 (55.7)	1625 (67.7)	
Female	2147 (44.3)	777 (32.3)	
BMI, kg/m^2^	24.50 ± 3.41	25.05 ± 3.25	<0.001
BMI category, n (%)			<0.001
Normal (<23)	1662 (34.3)	632 (26.3)	
Pre-obese (23–24.9)	1175 (24.2)	596 (24.8)	
Obese (≥25)	2012 (41.5)	1174 (48.9)	
Hypertension, n (%)	2649 (54.6)	1513 (63.0)	<0.001
Fasting plasma glucose, mg/dL	102.56 ± 20.55	107.86 ± 24.36	<0.001
HbA1c, %	5.77 ± 0.72	5.95 ± 0.87	<0.001
Glycemic status			<0.001
Normal (HbA1c ≤ 5.6)	2684 (55.4)	1078 (44.9)	
Prediabetes (5.7–6.4)	1670 (34.4)	923 (38.4)	
Diabetes (≥6.5)	495 (10.2)	401 (16.7)	
Total cholesterol, mg/dL	196.30 ± 42.77	193.34 ± 44.72	0.007
Triglyceride, mg/dL	101.00 (72.00–148.00)	112.00 (80.00–162.00)	<0.001
Hypertriglyceridemia, n (%)	1188 (24.5)	720 (30.0)	<0.001
Low HDL-C (M < 40, F < 50), n (%)	655 (13.5)	404 (16.8)	<0.001
High LDL-C (≥130), n (%)	1877 (38.7)	897 (37.3)	0.26
TyG index	8.59 ± 0.60	8.72 ± 0.61	<0.001
Metabolic syndrome, n (%)	1315 (27.1)	886 (36.9)	<0.001

BMI, body mass index; SBP, systolic blood pressure; HDL-C, high-density lipoprotein cholesterol; LDL-C, low-density lipoprotein cholesterol; FPG, fasting plasma glucose; HbA1c, glycated hemoglobin; TG, triglyceride; TyG, triglyceride–glucose index; SD, standard deviation; IQR, interquartile range.

**Table 2:** Clinical characteristics and adenoma prevalence across TyG index quartiles.

Variable	Q1	Q2	Q3	Q4	*p*-Value	*p* Trend
TyG range	7.00–8.19	8.19–8.59	8.59–9.01	9.01–12.03		
Adenoma, n (%)	476 (26.3)	580 (32.0)	644 (35.5)	702 (38.7)	<0.001	<0.001
Large adenoma (≥10 mm), n (%)	41 (2.3)	75 (4.1)	71 (3.9)	98 (5.4)	<0.001	<0.001
BMI, kg/m^2^	23.01 ± 2.99	24.22 ± 3.02	25.31 ± 3.25	26.17 ± 3.33		<0.001
Hypertension, n (%)	801 (44.2)	982 (54.1)	1120 (61.8)	1259 (69.5)	<0.001	<0.001
Diabetes (HbA1c ≥ 6.5%), n (%)	76 (4.2)	149 (8.2)	232 (12.8)	439 (24.2)	<0.001	<0.001
Metabolic syndrome, n (%)	89 (4.9)	243 (13.4)	544 (30.0)	1325 (73.1)	<0.001	<0.001

TyG, triglyceride–glucose index; Q, quartile; BMI, body mass index; HbA1c, glycated hemoglobin; SD, standard deviation.

**Table 3:** Univariable logistic regression for colorectal adenoma.

Variable	OR (95% CI)	*p*-Value
Age (per 10 yr)	1.49 (1.43–1.57)	<0.001
Male (vs. female)	1.66 (1.50–1.84)	<0.001
BMI (per 1 kg/m^2^)	1.05 (1.03–1.06)	<0.001
Obesity (BMI ≥ 25)	1.35 (1.22–1.49)	<0.001
SBP (per 10 mmHg)	1.17 (1.13–1.21)	<0.001
Hypertension	1.41 (1.28–1.56)	<0.001
FPG (per 10 mg/dL)	1.11 (1.09–1.14)	<0.001
HbA1c (per 1%)	1.33 (1.25–1.42)	<0.001
Diabetes (HbA1c ≥ 6.5%)	1.76 (1.53–2.03)	<0.001
TG (per 50 mg/dL)	1.08 (1.05–1.11)	<0.001
HDL-C (per 10 mg/dL)	0.85 (0.82–0.88)	<0.001
LDL-C (per 10 mg/dL)	0.98 (0.97–1.00)	0.019
Metabolic syndrome	1.57 (1.42–1.74)	<0.001
TyG (per 1-SD increase)	1.25 (1.19–1.31)	<0.001
TyG ≥ median	1.44 (1.30–1.59)	<0.001
TyG Q2 (vs. Q1)	1.32 (1.14–1.52)	<0.001
TyG Q3 (vs. Q1)	1.55 (1.34–1.79)	<0.001
TyG Q4 (vs. Q1)	1.78 (1.54–2.05)	<0.001

**Table 4:** Multivariable logistic regression models for the presence of colorectal adenoma: ORs for TyG (per 1-SD increase) with stepwise adjustment.

Variable	Model 1 (Crude)	P1	Model 2	P2	Model 3	P3	Model 4 (Full)	P4
TyG (per 1-SD)	1.25 (1.19–1.31)	<0.001	1.20 (1.14–1.27)	<0.001	1.16 (1.10–1.23)	<0.001	1.13 (1.06–1.20)	<0.001
Age (per 10 yr)	—	—	1.52 (1.45–1.59)	<0.001	1.52 (1.44–1.59)	<0.001	1.50 (1.43–1.58)	<0.001
Male	—	—	1.59 (1.43–1.77)	<0.001	1.55 (1.39–1.73)	<0.001	1.53 (1.36–1.71)	<0.001
BMI	—	—	—	—	1.03 (1.01–1.04)	0.003	1.02 (1.01–1.04)	0.011
Hypertension	—	—	—	—	1.08 (0.97–1.20)	0.165	1.08 (0.97–1.21)	0.141
HDL-C (per 10 mg/dL)	—	—	—	—	—	—	0.97 (0.93–1.01)	0.120
LDL-C (per 10 mg/dL)	—	—	—	—	—	—	1.01 (0.99–1.02)	0.314
Diabetes (HbA1c ≥ 6.5%)	—	—	—	—	—	—	1.19 (1.02–1.39)	0.030

**Table 5:** Fully adjusted multivariable model for large adenoma (≥10 mm).

Variable	OR (95% CI)	*p*-Value
TyG (per 1-SD)	1.25 (1.09–1.43)	0.001
Age (per 10 yr)	1.30 (1.15–1.46)	<0.001
Male	1.37 (1.04–1.80)	0.024
BMI	0.96 (0.92–1.00)	0.060
Hypertension	1.21 (0.93–1.57)	0.153
HDL-C (per 10 mg/dL)	0.89 (0.80–0.99)	0.028
LDL-C (per 10 mg/dL)	0.99 (0.96–1.03)	0.653
Diabetes (HbA1c ≥ 6.5%)	1.04 (0.74–1.46)	0.822

**Table 6:** Subgroup analysis: adjusted OR for adenoma per 1-SD increase in TyG index.

Subgroup	Cases/N	OR (95% CI)	*p* Interaction
Overall	2402/7251	1.15 (1.08–1.22)	
Sex			0.837
Male	1625/4327	1.16 (1.08–1.24)	
Female	777/2924	1.13 (1.01–1.26)	
Age			0.169
<50 yr	495/2339	1.15 (1.03–1.28)	
≥50 yr	1907/4912	1.13 (1.06–1.21)	
BMI			0.938
Non-obese (<25)	1228/4065	1.14 (1.05–1.24)	
Obese (≥25)	1174/3186	1.17 (1.08–1.27)	
Hypertension			0.176
Normotensive	889/3089	1.08 (0.98–1.19)	
Hypertensive	1513/4162	1.19 (1.11–1.28)	
Diabetes			0.634
No diabetes	2001/6355	1.13 (1.05–1.20)	
Diabetes	401/896	1.16 (1.01–1.34)	
Metabolic syndrome			0.557
Absent	1516/5050	1.11 (1.02–1.20)	
Present	886/2201	1.13 (1.02–1.26)	

## Data Availability

The data presented in this study are available on reasonable request from the corresponding author. The data is not publicly available because it contains information that could compromise the privacy of research participants.

## Abbreviations

The following abbreviations are used in this manuscript:

AUC	Area under the receiver-operating-characteristic curve
BMI	Body mass index
CI	Confidence interval
CRC	Colorectal cancer
FPG	Fasting plasma glucose
HbA1c	Glycated hemoglobin
HDL-C	High-density lipoprotein cholesterol
HOMA-IR	Homeostatic model assessment of insulin resistance
IQR	Interquartile range
LDL-C	Low-density lipoprotein cholesterol
NCEP-ATP III	National Cholesterol Education Program Adult Treatment Panel III
NPV	Negative predictive value
OR	Odds ratio
PI3K/Akt	Phosphoinositide 3-kinase/protein kinase B
PPV	Positive predictive value
ROC	Receiver-operating-characteristic
SD	Standard deviation
TG	Triglyceride
TyG	Triglyceride–glucose
